# Community-Based Decision Making and Priority Setting Using the R Software: The Community Priority Index

**DOI:** 10.1155/2015/347501

**Published:** 2015-02-26

**Authors:** Hamisu M. Salihu, Abraham A. Salinas-Miranda, Arnut Paothong, Wei Wang, Lindsey M. King

**Affiliations:** ^1^Department of Family and Community Medicine, Baylor College of Medicine, 3701 Kirby Drive, Suite 600, Houston, TX 77098, USA; ^2^REACHUP, Inc., 2902 N. Armenia Avenue, Suite 100, Tampa, FL 33607, USA; ^3^Department of Epidemiology and Biostatistics, College of Public Health, University of South Florida, 13201 Bruce B. Downs Boulevard, MDC 56, Tampa, FL 33612, USA

## Abstract

This paper outlines how to compute community priority indices in the context of multicriteria decision making in community settings. A simple R function was developed and validated with community needs assessment data. Particularly, the first part of this paper briefly overviews the existing methods for priority setting and reviews the utility of a multicriteria decision-making approach for community-based prioritization. The second part illustrates how community priority indices can be calculated using the freely available R program to handle community data by showing the computational and mathematical steps of CPI (Community Priority Index) with bootstrapped 95% confidence intervals.

## 1. Introduction

Providing public health practitioners and community development advocates with reliable measures for priority setting is a necessary step to foster accountability of the decision-making process in community settings. Community engagement is considered to be the pivotal element for a successful community-based organization [1, 2], particularly during the implementation of needs assessment projects and the selection of priorities for community action. By involving all relevant community stakeholders in the development of community action plans, community-based organizations not only ensure an equitable decision-making process but also enhance the cultural acceptability of interventions [3–5]. Although priority setting is an essential decision-making step for community-based organizations and participatory action research, there is little guidance on how to approach priority setting with quantifiable indicators while adopting community engagement principles [6].

Techniques for prioritization in community settings range from simple to more complex consensus building techniques, such as straight voting, weighted voting, nominal group technique, consensus panels, focus groups, Delphi technique, and others. The simplest form of community prioritization often occurs in town hall meetings or board meetings using simple voting, which typically implies giving each stakeholder the opportunity to vote on a list of issues. A variation of simple voting is the assignment of a certain number of votes to each stakeholder (e.g., 3 votes) and then sorting ideas or ranking to select the top one. Although democratic, this form of prioritization can only be used when the number of choices is small, and it is sensitive to issues of representativeness and generalizability. However, such a decision-making approach becomes increasingly cumbersome and impractical as the number of priorities increases. Another issue with straight voting is that it takes up only the majority of opinions and may inadvertently alienate a minority group, which can result in detrimental consequences for the community partnership and the engagement process. Some community advocates also use weighted voting, in which stakeholders assign different points (e.g., 1 = low importance, 2 = medium importance, and 3 = high importance) to a list of community issues in order to rank the items posteriorly. Although such a process tends to be more equitable, this method assumes that decision-makers are capable of mentally assigning reliable weights of diverse issues. This assumption is impractical because unguided stakeholders will reflect their personal preference and there is no guarantee that they will use uniform and consistent defensible criteria for prioritization every time they vote.

Because of some limitations of simple weighted methods, community development scholars recommend that voting methods are complemented with group discussions toward building a consensus to capture the community perspective rather than personal references. The two most frequently used consensus building methods are the nominal group technique (NGT) [7, 8] and Delphi technique [9–15]. By combining ranking procedures and participatory discussions, these techniques can be very effective in gathering consensus across diverse groups of stakeholders in a democratic and unbiased manner [10–12, 14–16]. The nominal group technique uses a one rank-ordered feedback, followed by a discussion that results in community consensus. However, a group of stakeholders that surpasses ten or twelve members cannot be easily managed and consensus may not be achieved. In contrast, the Delphi technique can encompass larger numbers of stakeholders and include several iterations of ranking and reranking (typically three or four) and consensus discussions. Because of its iterative nature and capacity to incorporate larger numbers of stakeholders, the Delphi technique is more robust than other methods. However, it can result in a lengthy process of several weeks or months. Because its implementation often requires the technical skills of highly skilled facilitators, the Delphi technique may be difficult to be implemented in community settings [9–11, 14, 15].

Community practitioners may also utilize qualitative techniques such as focus groups or key informant interviews. Qualitative methods provide credible and transferrable contextual data but in order to obtain generalizability, we need to implement mixed methods approaches. The value of qualitative techniques lies in gathering culturally relevant and richly experiential data, whereas quantitative techniques can complement qualitative findings (e.g., focus group themes) to generate measureable indicators that are comparable across settings (priority scores), different populations (mothers, children, and different geographical areas), and over different periods of time (longitudinal/repeated measures assessment).

None of the techniques mentioned previously explicitly differentiate between criteria of importance and changeability for the issues under consideration. For instance, stakeholders may decide to address highly important topics that are very difficult to change, which will result in projects that are ineffective and with discouraging results for the community. Conversely, stakeholders may decide to prioritize those issues that are highly changeable, but those issues may be of relatively low importance. The later situation would result in inefficient use of the scarce resources. We consider that community-based organizations must aim to address highly changeable and highly important issues. Since there is little guidance for community-based organizations on how to integrate these two criteria, we felt the need to develop a combined measure that indicates priority based on both importance and changeability.

## 2. The Need for the Development of the Community Priority Index

There is a clear need for quantifiable indicators for priority setting that integrate importance and changeability and permit cross settings comparisons but at the same time permit the wide participation of community stakeholders. Therefore, we developed the Community Priority Index using the following stepwise approach.

### 2.1. Adoption of Multiple Decision-Making Criteria

The adoption of a multicriteria decision-making approach in community-based priority setting has been widely recommended [17–19]. We recommend that decision-makers at least adopt a minimum of two criteria: importance and changeability. We selected importance and changeability because these are commonly used decision criteria in community-based program planning, but their utilization remains based on judgment and it is difficult to replicate due to the lack of quantifiable and comparable measures [20, 21]. Importance pertains to how relevant the issue was to the community context, which could be based on the magnitude of a particular problem (e.g., how prevalent, how much healthcare cost burden, or contribution to life expectancy or quality of life, or how relevant the problem is for the community under discussion). It is important to note that decision-makers can adopt separate importance criteria, such as importance based on cost, importance based on number of people dying from associated diseases, and importance based on impact on community quality of life. To simplify the present analysis, we use only one overall importance criterion. The second criterion we recommend is changeability, which refers to how easily the issue could be changed in the community if a designated intervention would be made available within the scope of a particular community-based organization.

### 2.2. Importance and Changeability Ratings for Each Decision-Making Criterion

Separate stakeholders' rating for the criteria of importance and changeability for each community issue must be identified, using weighted numerical scores (from 1 = low to 3 = high). We used a 3-point Likert-type scale, as follows: for importance: 0 = not important, 2 = intermediate importance, and 3 = very important; for changeability: 0 = not changeable, 2 = intermediate changeability, and 3 = highly changeable.

The mathematical computation consists of the following steps.

 Let *N*_*I*_ be a number of stakeholders (interviewers or decision-makers); each interviewer will prioritize the *N*_*q*_ questions (issue) using *N*_*c*_ criteria for each question.
 Let *x*_*iqc*_ be a *k*-Likert scale representing the score of the *c*th criteria of the *q*th question of the *i*th interviewer; thus, 1 ≤ *x*_*iqc*_ ≤ *k* for all *i*,  *q*,  *c*.

### 2.3. Computation of Item Average Scores by Importance and by Changeability

An important caveat is that, in community settings, it is typical to get an unequal number of participants' responses per item. This situation occurs because some items are responded by all members, while a few are responded by only a subset of the members (e.g., some stakeholders may leave blank spaces  for abstaining from voting or just missingness at random). The use of simple sum of item scores is inappropriate in such a situation. Thus, we used the arithmetic mean or average and computed mean importance scores and mean changeability scores. Accordingly, each sum of item scores was divided by the number of respondents for the particular item, which resulted in the item mean importance as well as item mean changeability. Forced responses are not recommended, since it may be perceived as coercion and a threat to the democratic process.

The mean of the *c*th criteria of the *q*th question, ${\overset{-}{x}}_{*qc}$, is calculated as follows:

(1) $$\begin{array}{c} {\overset{-}{x}}_{*qc}=n_{I}^{-1}\overset{n_{I}}{\underset{i=1}{\sum }}x_{iqc}. \end{array}$$

### 2.4. Multiplication of Mean Importance and Mean Changeability Item Scores to Generate a Summary Statistic That We Refer to Here as Community Priority Index (CPI)

We used the following formula: CPI = Mean  Importance∗Mean  Changeability. A single summary index was computed for each item or issue which integrated both perceived importance and perceived changeability, with higher values indicating higher priority.

The CPI is the product of the mean of the *c*th criteria of the *q*th question, calculated as follows:

(2) $$\begin{array}{c} {\text{CPI}}_{q}=\overset{n_{c}}{\underset{c=1}{\prod }}{\overset{-}{x}}_{*qc}=\overset{n_{c}}{\underset{c=1}{\prod }}\left(n_{I}^{-1}\overset{n_{I}}{\underset{i=1}{\sum }}x_{iqc}\right). \end{array}$$

### 2.5. Stratification by Target Population

If two or more subpopulations are targeted, then we recommend the stratification of CPI scores by types of population to identify priorities for action. This is a final step in which the issues are organized by type of population and ordered in descending fashion to identify the top highly important and highly changeable issues by target population. In this manner, community stakeholders will be able to better determine the scope of community-based strategies and to allocate project resources more effectively. Notably, the process is systematic and democratic, from beginning to the final selection of top priorities and by diverse populations separately (e.g., women, children, men, and the elderly).

### 2.6. Construction of 95% Confidence Intervals and Bootstrapping

This step of CPI pertains to the evaluation of the precision of asymptotic approximations in small samples, which is an important step if the number of stakeholders is relatively small (e.g., less than 30). For this purpose, we constructed 95% confidence intervals.

The lower bound (LB) of CPI_*q*_ can be calculated by assuming *x*_*iqc*_ = 1 for all *i*,  *q*,  *c*. Thus, LB of CPI is LB_CPI_ = ∏_*c*=1_^*n*_*c*_^(*n*_*I*_^−1^∑_*i*=1_^*n*_*I*_^1) = ∏_*c*=1_^*n*_*c*_^(*n*_*I*_^−1^ *n*_*I*_) = ∏_*c*=1_^*n*_*c*_^1 = 1. Similarly, the upper bound (UB) of CPI_*q*_ can be calculated by assuming *x*_*iqc*_ = *k* for all *i*,  *q*,  *c*. Thus, UB of CPI is UB_CPI_ = ∏_*c*=1_^*n*_*c*_^(*n*_*I*_^−1^∑_*i*=1_^*n*_*I*_^ *k*) = ∏_*c*=1_^*n*_*c*_^(*n*_*I*_^−1^ *n*_*I*_ *k*) = ∏_*c*=1_^*n*_*c*_^ *k* = *k* ^*n*_*c*_^. That is, the range of CPI_*q*_ is [1, *k* ^*n*_*c*_^].

It is important to highlight that the traditional confidence interval estimator that is based on the normal assumption of the sampling distribution cannot be used with small samples [22]. To overcome this limitation, we complemented the classic analysis with bootstrap methods to construct 95% confidence intervals [23]. Bootstrapping samples were created by ten thousand samples with replacement from the original dataset. The 2.5th percentile and 97.5th percentile are represented as the 95% CI of CPI. In bootstrapping, data collected for a single experiment is used to simulate what the results would have been if the experiment was repeated over and over with new samples (e.g., sampling with replacement from the original dataset). Specifically, we used bootstrap samples to estimate the mean score of 3-point Likert-type scaled items and their 95% confidence interval. By using bootstrap methods the distribution of the data normalizes permitting the use of the mean as a reference cut point [23]. Therefore, we generated via computer program (S+ 8.2) 5000 bootstrap samples of community stakeholders ratings [24]. The following algorithm was used to generate the bootstrap samples.

1. We constructed an empirical distribution function, $\hat{F}$, from the observed data. $\hat{F}$ places probability 1/*n* on each observed data point *x*_1_, *x*_2_,…, *x*_*n*_  (*n* = 6).
2. We then drew a bootstrap sample *X*_1_^*^, *X*_2_^*^,…, *X*_n_^*^ of size 6 with replacement from $\hat{F}$. The mean of this bootstrap sample was calculated achieving a normally distributed population.
3. Step 2 was repeated 10,000 times. The percentile method was used to compute a 95% confidence interval around the mean by ranking the bootstrap sample means and then selecting the 2.5 percentile as the lower confidence limit and the 97.5 percentile as the upper confidence limit. In other words, the lower bound value is the least CPI score possible within the 95% confidence interval, while the upper bound value is the highest possible CPI score within the interval. In this regard, the mean value of CPI scores for each issue represents the group consensus, whereas the width of 95% confidence intervals indicates the range of agreement.

### 2.7. Standardization of CPI Scores

Up to this point, CPI results are still scale-dependent and lack comparability potential with other community settings if different Likert-type scales are used (e.g., 3-point scale versus 5-point scale or 7-point scale). Comparability becomes particularly important for nation-wide programs or coalitions that have local or county chapters. Thus, we standardized each CPI indicator to have a range from 0 to 1 by applying the following conceptual formula:

(3) Standardized  CPI  =Actual  value−Lower  bound  valueUpper  bound  value−Lower  bound  value.

Accordingly, the mathematical formula standardizes each CPI indicator to have a range from 0 to 1 by applying the following formula:

(4) $$\begin{array}{c} {s\text{CPI}}_{q}=\frac{{\text{CPI}}_{q}-{\text{LB}}_{\text{CPI}}}{{\text{UB}}_{\text{CPI}}-{\text{LB}}_{\text{CPI}}}=\frac{{\text{CPI}}_{q}-1}{k^{n_{c}}-1}. \end{array}$$

**Figure 1:**
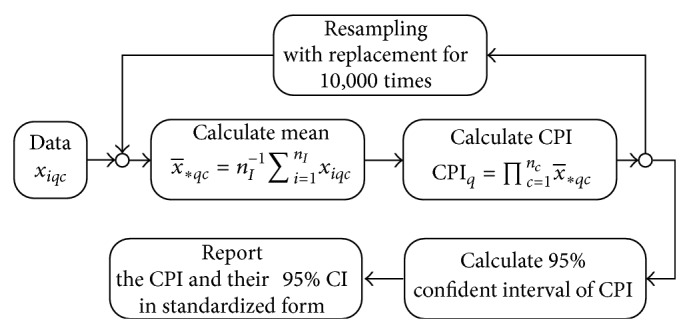
Mathematical computational process for the community priority indices.

Given the above formula, the CPI can only range from 0 to 1 and it is scale-free, which now permits comparisons across different studies and populations. The entire computational process of the CPI can be summarized in Figure 1.

## 3. R Code for Computation Process of CPI

In order to make the benefits of the CPI freely available to community practitioners, we developed a statistical program using the software R, which is free and widely available for download through the worldwide web. For any R users, we provide the R code for CPI computation.

### 3.1. Data Preparation

1. Its extension is  .csv.
2. The 1st column is ID of interviewee.
3. The 2nd column is question number.
4. The 3rd is *k*-Likert scale for the first criteria (importance).
5. The 4th is *k*-Likert scale for the second criteria (changeability).

### 3.2. R Functions

#### 3.2.1. LikertCheck Function

This function will return the data replacing all inappropriate scores that are outside the appropriate range (1 ≤ *x*_*iqc*_ ≤ *k*) with NA (see Algorithm 1).

#### 3.2.2. CPICal Function

This function will return the CPI of data (see Algorithm 2).

#### 3.2.3. CI95.CPI Function

This function will resample the data with replacement and calculates its CPI for 10,000 times (default). It will return the 2.5th and 97.5th percentile of 10,000 CPI representing the 95% confidence interval of CPI (see Algorithm 3).

#### 3.2.4. CPIReport Function

This function will calculate the CPI, its 95% CI, and their standardized form (see Algorithm 4).

Table 1 depicts a hypothetical set of scenarios where CPI was calculated for three populations that had unbalanced lists of issues, which is likely a situation that will arise in diverse community contexts. For this exercise, we derived CPI scores from a community needs assessment data conducted as part of previous R24 grant (i.e., purpose was to identify the top health priorities for community action to eliminate disparities in maternal and child health populations). However, we omitted the names and specific populations to permit a standard presentation of the CPI computation. The following cut-off points for prioritization were adopted for CPI scores, based on combined importance and changeability: values <0.3 were considered low priority, 0.3–0.7 intermediate priority, and >0.7 high priority. We recognize that these cut-off points are arbitrary, and practitioners may need to exercise more strict and narrow ranges according to their needs.

It can be appreciated in Table 1 that, for hypothetical population A, there were four standard CPI scores with values higher than 0.70, which indicates those issues were perceived as highly important and highly changeable priorities. However, only the top issue had a lower bound 95% higher than 0.70 (CPI for A1 = 0.85; 95% CI: 0.70, 1.00), which indicated a stronger consensus among decision-makers. In contrast, for issues A2 and A3, the standard CPI scores were higher than 0.70, but the respective 95% CI was wider (opinions vary more). Therefore, decision-makers can clearly select one top issue for population A and be also confident that there is a strong consensus that this issue is highly important and changeable.

For comparison purpose, we included subpopulations B and C, which had their own list of issues that were ranked and CPI scores computed. The main reason for adding populations is to illustrate how CPI could help prioritize populations too by carefully analyzing the CPI scores. In Table 1, it can be appreciated for population B that three issues presented standard CPI scores higher than 0.70. However, when looking at their 95% CI, all three issues had lower bounds with less than 0.70. This may indicate that decision-makers were less consistent regarding their assessment of importance and changeability or that there is less agreement in the selection as top issue. Since the CPI scores are standardized, practitioners can compare across populations and evaluate the consistency of the evidence across populations. In this particular exercise, decision-makers had stronger consensus regarding the importance and changeability of A1 issue than for B1 (CPI = 0.87; 95% CI = 0.69, 1.00) or C1 issues (CPI = 0.88; 95% CI = 0.69, 1.00). Therefore, if practitioners need to only prioritize one issue due to scarce resources, they can confidently select A1 as their top more efficient way to allocate resources. However, depending on availability of resources and programmatic focus, they can also justify additional issues that were also important and changeable, but for which consensus was weaker.

## 4. Conclusion

This study examined the development of a quantifiable indicator for priority setting that we have referred to as the Community Priority Index (CPI). We utilized two criteria (importance and changeability), as well as stratified by subpopulations. We consider that our CPI is a new measure that fosters the accountability of decision making, while flexibly allowing for the application of diverse participatory methodologies. For example, the list of issues that must be prioritized can be generated through nominal groups, focus groups, community surveys, or even expert opinions. However, if the CPI is used in conjunction with democratic participation and adequate community engagement processes, the decision of prioritizing will explicitly incorporate aspects of relevance and changeability. Thus, rather than replacing other methods, we recommend the CPI as an additional tool that can be incorporated to community-based efforts for priority setting, if acceptable and relevant for the community and project context.

Our R code can be easily modified to suit the needs of different projects and help decision-makers in the application of multiple decision-making criteria. Notably, since the R package is free of cost and available for multiple operating systems, community-based organizations from all over the world can use the CPI to foster accountability in the selection of priorities for action. We recommend that practitioners use the 95% CIs for the final assessment of top priorities and weight their decision against willingness to pay and budget allocations, as well as ethical factors, social justice, and cultural competence of interventions. In particular, the bootstrapped 95% CI provides an opportunity to assess the precision of the CPI scores with even small samples of decision-makers. While the mean CPI score for a particular issue (standardized CPI) indicates the level of group consensus on importance and changeability, the width of the 95% CI indicates the scope of agreement or group consensus. Wider 95% CI indicates that group opinions were more diverse. Diversity of opinion among group leaders may directly indicate more controversy in the community since their opinion represents the community perspective to a certain extent. Using our recommended cut-off values, practitioners can choose priorities that were highly important and highly changeable, as well as selecting those for which there is greater agreement. CPI is presented here as a tool in tandem with advancing the science of mixed methods analysis by incorporating both qualitative and quantitative data in the way to enhance precision in the assessment of group consensus, as well as enhanced interpretation of the multiple decision-making criteria. In this context, researchers wishing to use the CPI must acknowledge the ecological and political nature of community-based participatory research by assessing individual opinions through adequate participatory methods. Indeed, the partnership synergy is an extremely important aspect of community-based organizations and participatory action research that describes how early disagreements and conflicts can produce more productive stakeholder interactions in the future. Thus, the CPI is not a replacement of stakeholders' consensus building discussions. Instead, the CPI is a way to implement multicriteria decision-making approach and to foster accountability of decisions.

## Figures and Tables

**Algorithm 1:**
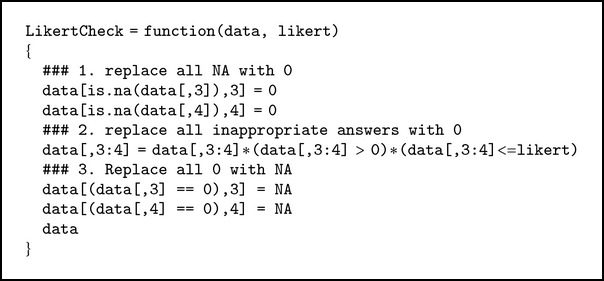


**Algorithm 2:**
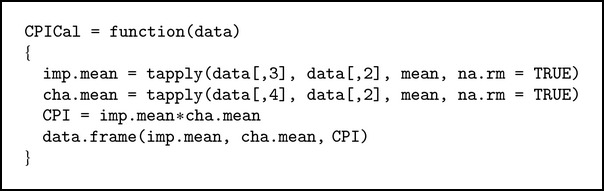


**Algorithm 3:**
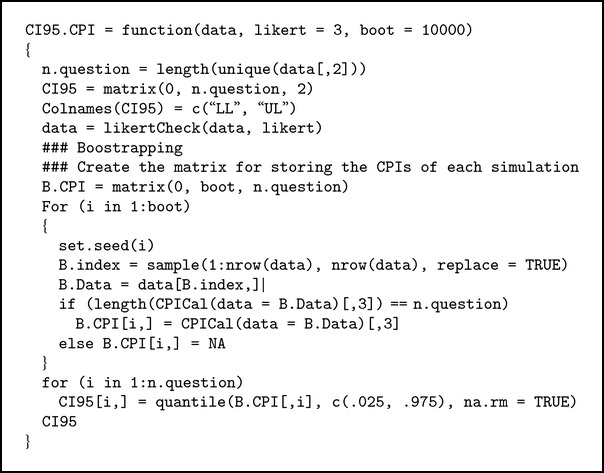


**Algorithm 4:**
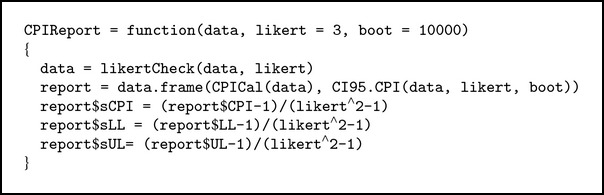


**Table 1:** Simulated 95% confidence intervals and standardized CPI scores.

Population	Issue	Observed CPI	95% bootstrap CI for CPI	Std. CPI	Std. 95% CI
Subpopulation A	A1	7.80	[6.60, 9.00]	0.85	[0.70, 1.00]
	A2	7.93	[6.50, 9.00]	0.87	[0.69, 1.00]
	A3	6.93	[5.50, 8.50]	0.74	[0.56, 0.94]
	A4	6.67	[5.42, 8.03]	0.71	[0.55, 0.88]
	A5	7.00	[6.00, 8.00]	0.75	[0.63, 0.88]
	A6	6.05	[4.95, 7.20]	0.63	[0.49, 0.78]
	A7	5.67	[4.25, 7.08]	0.58	[0.41, 0.76]
	A8	5.50	[4.50, 6.00]	0.56	[0.44, 0.63]
	A9	3.24	[1.80, 5.28]	0.28	[0.10, 0.54]

Subpopulation B	B1	7.93	[6.50, 9.00]	0.87	[0.69, 1.00]
	B2	7.37	[5.87, 8.50]	0.80	[0.61, 0.94]
	B3	6.61	[5.42, 8.00]	0.70	[0.55, 0.88]
	B4	6.00	[4.50, 7.50]	0.63	[0.44, 0.81]
	B5	5.83	[4.67, 7.11]	0.60	[0.46, 0.76]
	B6	5.50	[4.00, 7.00]	0.56	[0.38, 0.75]
	B7	4.80	[3.36, 6.24]	0.48	[0.30, 0.66]

Subpopulation C	C1	8.03	[6.67, 9.00]	0.88	[0.69, 1.00]
	C2	7.50	[6.50, 8.50]	0.81	[0.69, 0.94]
	C3	7.00	[5.00, 8.50]	0.75	[0.50, 0.94]
	C4	6.61	[5.33, 8.00]	0.70	[0.54, 0.88]
	C5	5.67	[4.25, 7.08]	0.58	[0.41, 0.76]

Note: CPI = Community Priority Index; Std. = standardized.

## References

[1] Israel B. A. (2005). *Methods in Community-Based Participatory Research for Health*.

[2] CDC/ATSDR Committee on Community Engagement (2011). *Principles of Community Engagement*.

[3] Moore T., Hennessy E. M., Myles J. (2012). Neurological and developmental outcome in extremely preterm children born in England in 1995 and 2006: the EPICure studies. *British Medical Journal*.

[4] Cook W. K. (2008). Integrating research and action: a systematic review of community-based participatory research to address health disparities in environmental and occupational health in the USA. *Journal of Epidemiology and Community Health*.

[5] Shalowitz M. U., Isacco A., Barquin N. (2009). Community-based participatory research: a review of the literature with strategies for community engagement. *Journal of Developmental and Behavioral Pediatrics*.

[6] Koch T., Kralik D. (2006). *Participatory Action Research in Health Care*.

[7] Gallagher M., Hares T., Spencer J., Bradshaw C., Webb I. (1993). The nominal group technique: a research tool for general practice?. *Family Practice*.

[8] van de Ven A., Delbecq A. L. (1971). Nominal versus interacting group processes for committee decision-making effectiveness. *Academy of Management Journal*.

[9] Delbecq A. L., van de Ven A. H., Gustafson D. H. (1975). *Group Techniques for Program Planning: A Guide to Nominal Group and Delphi Processes*.

[10] Hasson F., Keeney S., McKenna H. (2000). Research guidelines for the Delphi survey technique. *Journal of Advanced Nursing*.

[11] Mitchell M. P. (1998). Nursing education planning: a Delphi study. *The Journal of nursing education*.

[12] Okoli C., Pawlowski S. D. (2004). The Delphi method as a research tool: an example, design considerations and applications. *Information and Management*.

[13] Rideout C., Gil R., Browne R. (2013). Using the Delphi and snow card techniques to build consensus among diverse community and academic stakeholders. *Progress in Community Health Partnerships: Research, Education, and Action*.

[14] Rowe G., Wright G. (1999). The Delphi technique as a forecasting tool: issues and analysis. *International Journal of Forecasting*.

[15] Synowiez B. B., Synowiez P. M. (1990). Delphi forecasting as a planning tool. *Nursing management*.

[16] Clayton M. J. (1997). Delphi: a technique to harness expert opinion for critical decision-making tasks in education. *Educational Psychology*.

[17] Baltussen R., Niessen L. (2006). Priority setting of health interventions: the need for multi-criteria decision analysis. *Cost Effectiveness and Resource Allocation*.

[18] Ryan M., Scott D. A., Reeves C. (2001). Eliciting public preferences for healthcare: a systematic review of techniques. *Health Technology Assessment*.

[19] Ryan M., Kinghorn P., Entwistle V. A., Francis J. J. (2014). Valuing patients' experiences of healthcare processes: towards broader applications of existing methods. *Social Science and Medicine*.

[20] Gielen A. C., McDonald E. M., Gary T. L., Bone L. R. (2008). Using the precede-proceed model to apply health behavior theories. *Health Behavior and Health Education: Theory, Research, and Practice*.

[21] Li Y., Cao J., Lin H., Li D., Wang Y., He J. (2009). Community health needs assessment with precede-proceed model: a mixed methods study. *BMC Health Services Research*.

[22] Hoyle R. H. (1999). *Statistical Strategies for Small Sample Research*.

[23] Efron B., Tibshirani R. (1994). *An Introduction to the Bootstrap*.

